# Draft genome sequence of multidrug-resistant *Escherichia coli* strain ASK-30 from wastewater in northern India

**DOI:** 10.1128/mra.00009-26

**Published:** 2026-03-16

**Authors:** Shilippreet Kour, Shilpa Sharma, Achhada Ujalkaur Avatsingh, Prem Prashant Chaudhary, Nasib Singh

**Affiliations:** 1 Department of Microbiology, Akal College of Basic Sciences, Eternal University 243975, Baru Sahib, Himachal Pradesh, India; 2 Laboratory of Clinical Immunology and Microbiology, Epithelial Therapeutics Unit, National Institute of Allergy and Infectious Disease, National Institutes of Health 2511, Bethesda, Maryland, USA; University of Maryland Baltimore, Baltimore, Maryland, USA

**Keywords:** whole genome sequencing, antibiotic resistance, *Escherichia coli*

## Abstract

We report the draft genome sequence of the multidrug-resistant *Escherichia coli* strain ASK-30 isolated from wastewater in a northern state in India. Its genome harbors *bla*_CTX-M-15_, *bla*_TEM-1_, and other antibiotic resistance genes.

## ANNOUNCEMENT

*Escherichia coli* is a gram-negative bacterium present in the guts of humans and animals and is now recognized as a significant contributor to the dissemination of antimicrobial resistance (1–3). Many strains of *E. coli* are reported to carry multiple antibiotic resistance genes imparting resistance to β-lactams and other antibiotic classes (4). With the rise in multidrug resistance in *E. coli* from clinical settings, aquatic environments, and veterinary samples, treatment challenges and higher mortality have been encountered. Here, we report the draft genome sequence of *E. coli* strain ASK-30 from Amritsar, Punjab, India.

*E. coli* ASK-30 was isolated from wastewater influent collected from the municipal sewer drain located in Amritsar (Latitude 31.620019*°,* Longitude 74.879776*°*), Punjab, India. Bacteria were isolated on EMB agar and MacConkey agar by incubation at 35°C–37°C for 24 h. *E. coli* ASK-30 was evaluated for its susceptibility to different antibiotic classes by Kirby-Bauer disc diffusion method in accordance with Clinical Laboratory Standards Institute (CLSI) guidelines (5) and Drieux et al. (6). The results were interpreted as resistant, intermediate, or susceptible according to the CLSI document M100 (2021).

The bacterial culture was cultivated in nutrient broth at 37°C for 24 h, followed by genomic DNA extraction using Xploregen DNA Kit (Xploregen, Bengaluru, India). The concentration of the extracted DNA was measured using a Qubit 3 fluorometer (Thermo Fisher Scientific, USA). For library preparation, 100 ng of gDNA was enzymatically fragmented to a target size of 200–300 bp. The resulting fragments underwent end repair and 3′-adenylation. Loop adapters were ligated and subsequently cleaved using the uracil-specific excision reagent enzyme. Following AMPure bead purification, DNA fragments were enriched via a 6-cycle PCR using NEBNext Ultra II Q5 master mix and Illumina primers. The final library was quantified using the Qubit 3 fluorometer, and fragment size analysis was performed (Agilent 2100 Bioanalyzer). Libraries were sequenced on an Illumina MiSeq Sequencing System (150 bp paired-end).

Sequencing quality was assessed with FastQC v0.12.1 (7), the draft genome was assembled using Unicycler v0.5.1 (8), and genome completeness was evaluated with BUSCO v6.0.0 (9). Contigs were ordered and oriented using the scaffolding tool Multi-CSAR v1.0 (10). The genome was annotated using the NCBI Prokaryotic Genome Annotation Pipeline v6.10 (11). Antibiotic resistance genes were identified using the Comprehensive Antibiotic Resistance Database (CARD v4.0.1; https://card.mcmaster.ca/), with only the perfect hits (100% identity and coverage) and strict hits (≥95% identity and ≥80%–90% coverage)being included in the analysis (12). Plasmid replicons were detected using PlasmidFinder v2.0.1 (13) and MGEfinder v1.0.3 (14). Virulence genes were identified using VirulenceFinder v2.0 (15). All the analyses were performed using default parameters unless specified otherwise. The results are summarized in Table 1.

**TABLE 1:** Genomic profile of *E. coli* strain ASK-30

Characteristic	Value
Draft genome size (bp)	4,997,113
N50/L50	252,455 (bp)/7
GC content (%)	54.1
Total reads	5,817,120
Number of contigs	78
Largest contig (bp)	545,782
Average contig length (bp)	64,065
Total genes	4,877
Total coding sequences (CDSs)	4,779
Genes (protein coding)	4,661
rRNA genes	3
tRNA genes	84
ncRNA genes	11
Genome accession no.	JBTAFD000000000
BioSample accession no.	SAMN48884702
BioProject accession no.	PRJNA1271716
SRA accession no.	SRR33827093
Phenotypic resistance	Ampicillin, piperacillin, cefazolin, cefuroxime, ceftriaxone, cefpodoxime, cefotaxime, cefprozil, streptomycin, tetracycline
Antibiotic resistance genes	*bla*_CTX-M-15_, *bla*_TEM-1_, *aph* (6)-Id_1, *aph*(3’’)-Ib_5, *sul*2, *tet*(A), *emr*Y, *emr*K, *qnr*S1, *ssemr*B, and *emr*R
Plasmid replicons	IncFIB(AP001918)_1 and IncFIB(K)
Virulence factors	*aslA, air*, *csgA, eilA, fdeC, hha, hlyE, iss, kpsE, kpsMII_K5, nlpI*, *terC, yehA, yehB, yehC,* and *yehD*

The draft genome of *E. coli* ASK-30 revealed the presence of multiple antibiotic resistance genes, plasmid replicons, and virulence factors. These findings provide insights into the antibiotic resistance mechanisms and highlight the role of wastewater in the transmission of antibiotic resistance.

## Data Availability

The draft genome assembly has been deposited in GenBank under the accession number JBTAFD000000000. The associated BioSample and BioProject identifiers are SAMN48884702 and PRJNA1271716, respectively. Raw sequencing reads are available in the Sequence Read Archive under the accession number SRR33827093.
